# Risks of concealing environmental degradation

**DOI:** 10.1111/cobi.70311

**Published:** 2026-05-12

**Authors:** Matt W. Hayward, Kaya Klop‐Toker, Salim Momtaz, Eliza Payne, Louisa Rebec, Robert J. Scanlon, Craig M. Shuttleworth, Nick Valentine, Andrea S. Griffin

**Affiliations:** ^1^ Centre for Conservation Science, College of Engineering, Science and the Environment University of Newcastle Callaghan Australia; ^2^ Centre for African Conservation Ecology Nelson Mandela University Port Elizabeth South Africa; ^3^ Mammal Research Institute University of Pretoria Tshwane South Africa; ^4^ Centre for Sustainable Development University of Liberal Arts Dhaka Bangladesh; ^5^ Environmental Leader JBS & G Pty Ltd St Ives NSW Australia; ^6^ School of Environmental and Natural Sciences Bangor University Bangor UK; ^7^ World Bank Consultant Valentine Environmental Manly NSW Australia

**Keywords:** buffer zones, environmental impacts, visual assessments, Evaluaciones visuales, impactos ambientales, zonas de amortiguación, 视觉评估, 视觉评估环境影响, 视觉评估缓冲区

## Abstract

Current practice seeks to conceal the visual impact of land‐use change (i.e., development). Six percent of development impact assessments in Australia and 14% of the World Bank's assessments recommend visual impact mitigation. This mitigation results in, for example, vegetated buffer strips alongside cleared agricultural areas and earthen mounds that block environmental degradation from view. These measures make living alongside such development more palatable, but they conceal its negative effects from the general public. The scale of the problem is global with evidence of mandated regulatory drivers for concealment from around the world. These mitigation measures limit the emotional impact of development on people, reducing their likelihood of implementing pro‐environmental actions. It is important for the populace to see the scale of human impacts on the environment to increase the likelihood of pro‐environmental behaviors and decisions that will help ease the crises facing the environment. We recommend overtly exposing the public to environmental degradation to improve understanding of environmental impacts, increase environmental knowledge, trigger pro‐environmental emotions and actions, and help recalibrate people's understanding of biodiversity loss. Policies and research into the effect of creative public imagery designed to improve communication into environmental destruction while minimizing habituation are important, and ensuring that the “greening” of gray infrastructure yields genuine biodiversity benefits is also critical.

## Abstract (es)

Riesgos por ocultar la degradación medioambiental

**Resumen:**

La práctica actual busca ocultar el impacto visual del cambio en el uso del suelo (es decir, el desarrollo urbanístico). El 6 % de las evaluaciones de impacto de proyectos de desarrollo en Australia y el 14 % de las del Banco Mundial recomiendan medidas de mitigación del impacto visual. Esta mitigación se traduce, por ejemplo, en franjas de vegetación a lo largo de las zonas agrícolas despejadas y en montículos de tierra que ocultan de la vista la degradación medioambiental. Estas medidas hacen más aceptable convivir con este tipo de desarrollo, pero ocultan sus efectos negativos al público en general. La magnitud del problema es global; existen factores normativos obligatorios que impulsan el ocultamiento en todo el mundo. Estas medidas de mitigación limitan el impacto emocional del desarrollo en las personas. Es importante que la población vea la magnitud de los impactos humanos sobre el medio ambiente para aumentar la probabilidad de comportamientos y decisiones proambientales que ayuden a aliviar las crisis a las que se enfrenta el medio ambiente. Recomendamos exponer abiertamente al público a la degradación ambiental para mejorar la comprensión de los impactos ambientales, aumentar el conocimiento sobre el medio ambiente, desencadenar emociones y acciones proambientales, y ayudar a recalibrar la comprensión de la gente sobre la pérdida de biodiversidad. Son importantes las políticas y la investigación sobre el efecto de las imágenes públicas creativas diseñadas para mejorar la comunicación de la destrucción ambiental y minimizar la habituación, al igual que garantizar que la ecologización de la infraestructura gris genere beneficios reales para la biodiversidad.

## 掩盖环境退化的风险

**【摘要】** 现行实践常常试图掩盖土地利用变化(即开发建设)带来的视觉影响。澳大利亚6%的开发影响评估报告以及世界银行14%的评估报告都建议采用视觉影响减缓措施。这些减缓措施的结果包括在已被清除的农业用地旁设置植被缓冲带, 以及堆砌土丘以遮挡环境退化的景象。这虽然能使人们更易接受在此类开发项目周边生活, 但却向公众隐瞒了开发建设的负面影响。该问题具有全球性, 世界各地均存在驱动这种隐瞒行为的强制性监管因素。这些减缓措施削弱了开发项目对人们的情感影响。让民众直观了解人类对环境的影响规模至关重要, 这有助于提高采取环保行为和决策的可能性, 从而缓解环境面临的危机。我们建议向公众公开展示环境退化的现象, 以增进对环境影响的理解、提升环保知识、激发环保情感与行动, 并帮助人们重新认识生物多样性丧失的严重性。我们提出, 未来的关键在于制定政策并开展研究, 探索如何通过创意性公共视觉呈现来更好地传达环境破坏信息, 同时尽量避免公众对此类信息的习惯化;此外, 还应确保灰色基础设施的绿化能真正带来生物多样性效益。**【翻译:胡怡思;审校:聂永刚】**

## INTRODUCTION

As visual animals, human sight greatly informs people about the environment (Morris, 1999; Politzer, 2008), and people more readily develop deep emotional attachments to things they can visualize (Pessoa & Adolphs, 2010; Tamietto & De Gelder, 2010). The importance of vision derives from its role in human evolution, from predator detection to communication and sexuality (Pessoa et al., 2014; Whittaker & O'Conaill, 1997; Yarber et al., 2009). Indeed, the area of the brain that processes visual information is larger than that for other senses (Kaas, 1989).

Vision is significant to humans because the sense is used in a multitude of ways to determine behaviors and decisions, including in subliminal ways. Visual imagery is the most effective form of advertising for sales (Prithvi & Dash, 2013; Rossiter & Percy, 1980). Health campaigns commonly employ visual imagery of human disease to discourage harmful behavior (Evans, 2006); imagery of animal mistreatment is a powerful means of mobilizing protest (Fernández, 2021); and in the environmental sector, individuals think more clearly about relevant issues when shown infographics incorporating imagery than when presented with text messages or illustrations (Lazard & Atkinson, 2015). Turtle conservationists have shown that high‐quality and emotive images of turtles influence the degree of pro‐conservation behaviors (Ison et al., 2024).

Yet, reliance on visual imagery can also cause humans to misconstrue important things, particularly when these occur at scales beyond the human ability to comprehend (Wilson et al., 1998). The vastness of the number of stars in the universe is difficult to directly imagine without analogous visual imagery of the number of grains of sand on all the beaches of the planet (Sagan, 1979). Without the image of a clock, the brevity of human life relative to the age of the planet would be difficult to grasp. Such issues of scale and longevity are also a common feature of global environmental degradation (Wilson et al., 1998).

**FIGURE 1:**
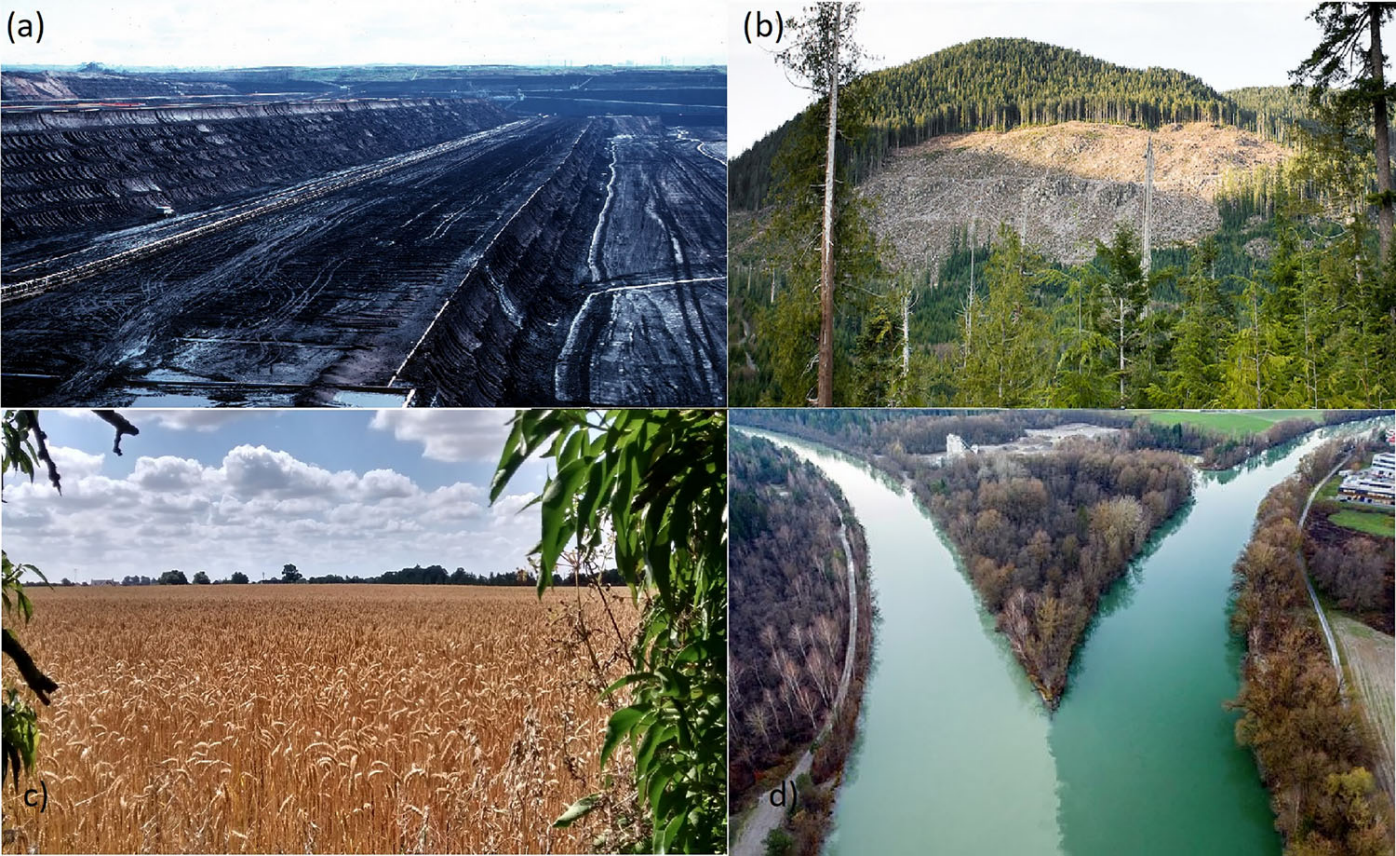
Examples of major developments that are routinely concealed from the general public by buffer zones or fences: (a) Morwell open‐cut coal mine in Victoria, Australia (CC BY 3.0), (b) clearcut old‐growth in the Gordon River Valley, British Columbia, Canada (T. J. Watt CC BY‐SA 3.0), (c) wheat monoculture (CC), and (d) toxic pollution in a river (CC).

Environmental degradation occurs globally and cumulatively at vast scales both spatially and temporally. It can take many forms, including the insidious and the visually striking. For example, open‐cut mining gouges deep holes in the earth; clearcutting forests leaves swathes of stumps, fallen logs, and machinery‐churned soil; agricultural cropping replaces native vegetation with vast monocultures; and pollution often stains rivers (Figure 1).

We aimed to show that concealment of environmental destruction foregoes an opportunity to help the broader public grasp the impact of environmental destruction on the planet and motivate environmentally friendly behavior and environmental advocacy. We gathered data on the frequency with which environmental damage is concealed from the public in impact assessment recommendations. Unless people can observe the scale of environmental degradation and destruction associated with the Anthropocene directly, they are unlikely to be concerned enough to drive change. In developing our arguments, we first considered the scale of the issue and the types of visual impact environmental destruction can take. Second, we examined how concealment of visual impact is often legislated or directed. Third, we examined the emotional impact of visual destruction on humans and proposed various psychological mechanisms by which this emotion could help drive behavioral change. Finally, we devised recommendations for policy changes and required research.

## SCALE OF THE PROBLEM

We reviewed 3664 major projects listed on the online planning portal for the Australian state of New South Wales (https://www.planningportal.nsw.gov.au/major‐projects/projects) from 2015 to 2025 (Appendix S1). We excluded urban local government areas because the natural visual landscape of these areas has already been heavily damaged (Appendix S2). We found 203 (5.5%) environmental impact assessments where the Secretary for the Department of Planning recommended visual impact assessments to take place. Ninety percent of these recommended visual impact mitigation measures (μ = 6.5, range 0–52), and 28% recommended using additional compensatory vegetation plantings to screen the visual impacts (Appendix S1). The key mitigation measures that arose involved using existing or additional vegetation to screen impacts (86% in total) and visual buffering by planting multirow vegetation screens (often 20‐ to 50‐m wide) along site boundaries or road frontages (e.g., Waterfall Way, Mitchell Highway; Appendix S1) to break up or block views of degradation. Specific design and siting recommendations were made in 70% of impact assessments, and these included placing bulky infrastructure (substations, sheds, stockpiles) in topographically low areas or behind hills, significant setbacks from public viewpoints and residences, reduced building height, increased articulation to create a slender profile, clustering components to minimize the development footprint, and muted palettes of nonreflective earth tones (e.g., eucalypt green, beige, muted browns) to help structures blend into the rural or natural background. Landowner agreements were also discussed to enable the installation of screening directly on adjacent private property if on‐site measures proved insufficient. Approximately, 10% of impact assessments had no specific visual impact mitigation measures recorded, often because the initial impact was assessed as negligible due to extreme distance from people or existing topography.

We performed a similar search with World Bank environmental and social impact assessments from 2024 to 2025 and found 190 impact assessment documents. Of these, 26 (13.7%) required explicit visual impact assessment, and these recommended a mean of 5 mitigation measures (range 0–36), with five projects requiring compensatory planting to obscure visual impacts (Appendix S3). The key mitigation recommendations from World Bank impact assessments were strict limits on the removal of existing natural vegetation, use of landscaping and vegetation cover to rehabilitate affected areas, use of visual barriers (e.g., trees), and siting of development farther away from sensitive areas to lower the project's aesthetic profile. Clearly, both in Australia and globally, concealing visual impacts is a common feature of impact assessments.

## VISUAL IMPACTS OF ENVIRONMENTAL DESTRUCTION AND DEGRADATION ON THE LANDSCAPE

**FIGURE 2:**
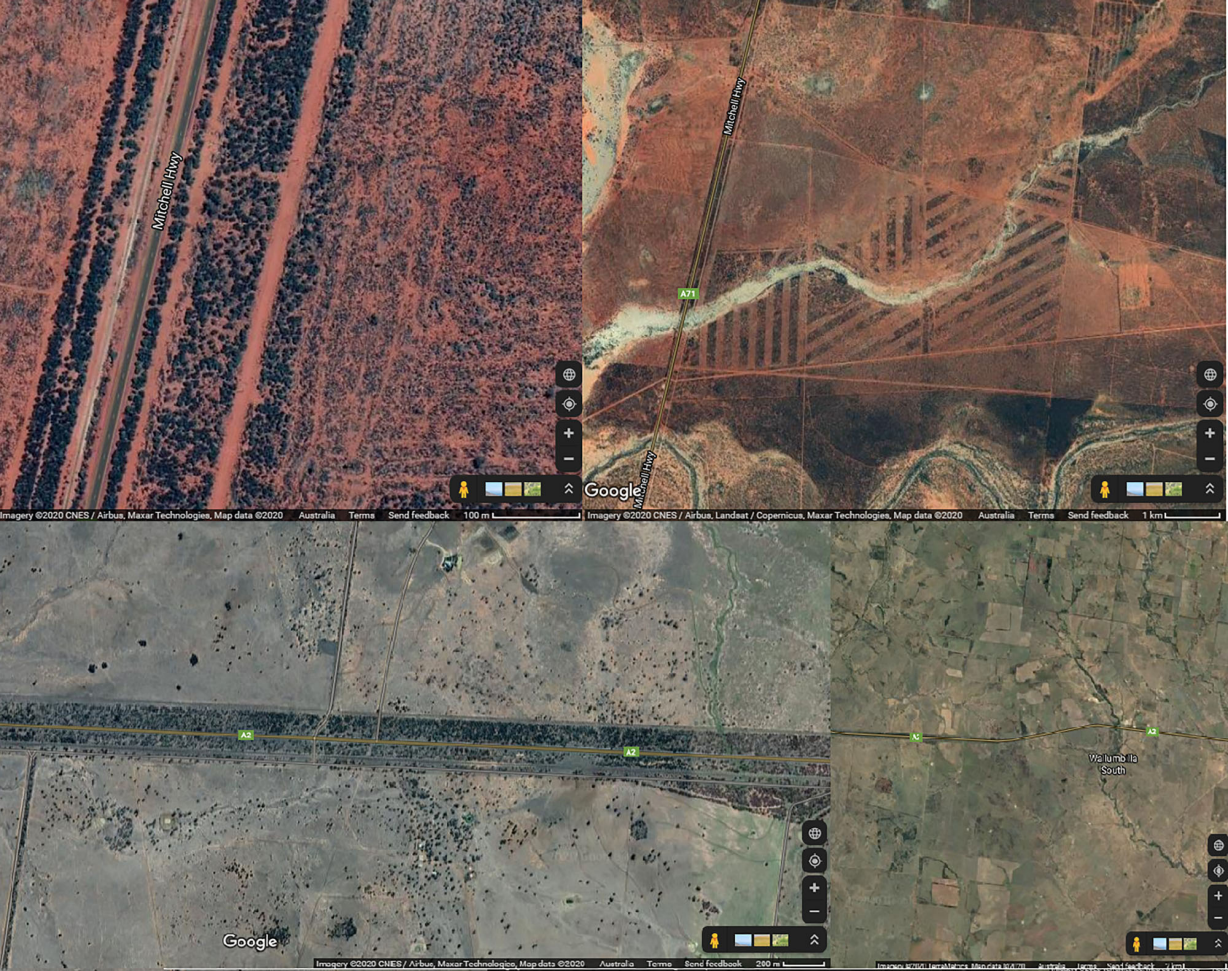
Narrow vegetation strips alongside highways in western Queensland, Australia, concealing the scale of the removal of vegetation (Google Maps).

**FIGURE 3:**
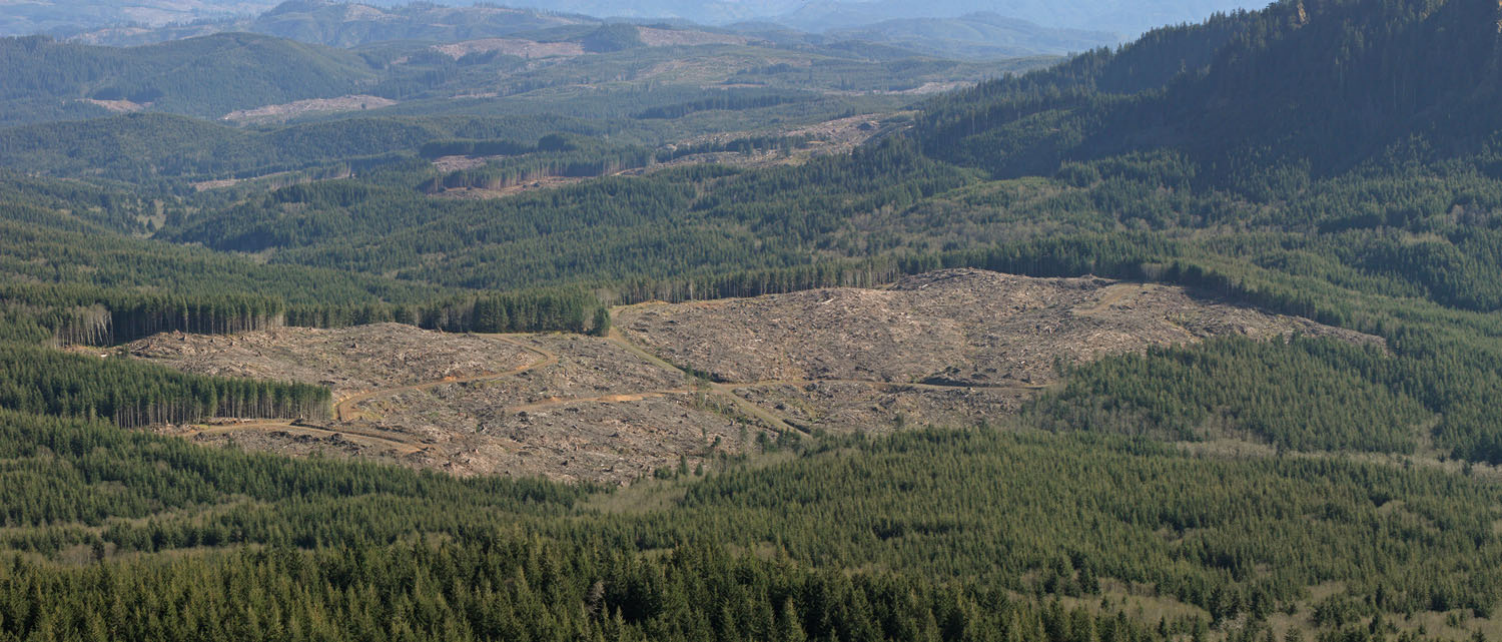
Clearcut area in the Lewis and Clark River Valley, Oregon. The large areas of forest around the cut conceal it from most viewpoints (CC BY‐SA 3.0).

**FIGURE 4:**
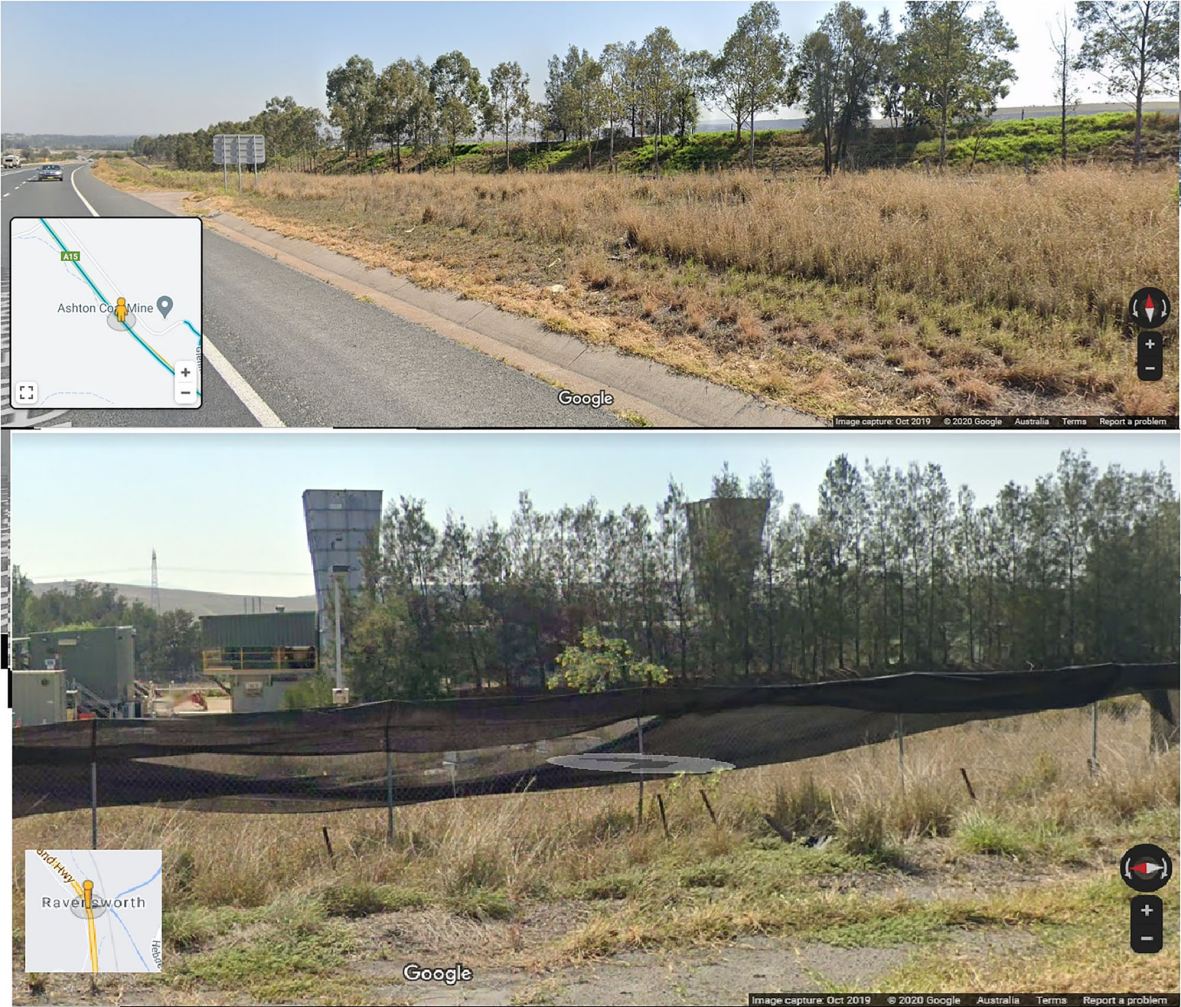
Visual barriers of different quality and efficacy at (top) the Ashton Coal Mine (earthen embankment with native tree canopy and likely exotic grass ground cover) and (bottom) a quarry, both of which are situated in the Hunter Valley of New South Wales, Australia (poorly maintained shade cloth) (Google Maps).

As illustrated above, visually striking environmental loss is routinely concealed from the general public. More broadly, verges of remnant native vegetation line highways through agricultural zones, and this can obscure the complete removal of native vegetation in adjacent extensive agricultural landscapes (Figure 2). Similarly, extensive forest clearance operations regularly occur in isolated areas or are buffered from view of roads or other high‐public‐use areas (Figure 3). Mines and quarries are routinely concealed from view with earthen, fabric, or vegetation barriers (Figure 4). Some of these activities may occur in remote areas simply because of their location; however, those activities that occur near areas frequented by people generally have their impacts concealed (Appendices S1 & S3). This may be because when environmental impacts are overtly displayed, they elicit powerful emotional responses in the observer that can drive behavioral change (Lertzman, 2010; Van Haaften & Van De Vijver, 1996).

## CURRENT LEGISLATION AND THE SPIRIT AROUND VISUAL IMPACT

The concealment of environmental devastation is often legislated (Appendix S4). There has been a well‐developed code for assessing the visual impacts of large structures on the landscape for decades (Appendix S4). This code was derived from Wilson (2002). More recently, the code has been adjusted to accommodate development associated with renewable energy facilities, including windfarms (Manchado et al., 2013) and photovoltaic stations (Chiabrando et al., 2011; Custódio et al., 2020). The underlying spirit of these assessments is that the visual impact on the landscape should be minimized, particularly in areas of high aesthetic and cultural value.

The preservation of areas of high aesthetic and cultural value is widely accepted as a valued component of the natural environment and is most readily achieved through land‐use planning. Frequently, however, visual impact from renewable energy projects in areas with lesser aesthetic value is cited by opponents as a determining factor in project approval. This is an extension of the NIMBY (not‐in‐my‐backyard) attitude whereby one's “backyard” extends to the entire viewshed that is somehow “owned” and carries with it veto power over the development. Although the views from a home will often be of considerable value (both aesthetically and monetarily), people do not have the power to protect their view as a legal, proprietary right. Although visual impact is a matter for consideration in planning decisions, even a devastating visual impact may be considered reasonable in the circumstances when considering the overall justification for a development (Lindsay Taylor Lawyers, 2015).

The same spirit of impact minimization is present in industries that destroy natural landscapes. Although minimizing visual impact of renewable energy structures to maximize public acceptance is in line with the need to move toward a sustainable future, we argue that similarly minimizing environmental destruction in this manner is detrimental to motivating behavioral change. By hiding destruction of the environment from the public eye, large profitable industries greatly contribute to major biodiversity losses without general public knowledge.

Many states and territories in Australia have some form of legal or policy instruments that direct proponents and developers to consider visual impacts of proposed development activities before commitments are made (Appendix S4), although these are not always clear. In New South Wales, landscape and visual impact assessments are required for developments considered significant by the state government approval agencies that are based on specific industry guidelines, such as large‐scale solar energy guidelines. Smaller local developments depend on local council guidelines within their development control plans that could relate to project size, class, or the visual character of the locality. For example, the Lake Macquarie Development Control Plan 2014 includes a “Scenic Values” section that outlines the types of developments that trigger visual impact assessment. Although these assessments consider the potential impacts of a proposed development on landscape quality and visual amenity, they can also be used to justify visually invasive developments through proposed mitigation measures, such as visual barriers of trees, fences, and walls.

In the United States, different jurisdictions have distinct laws and directives of visual impact assessment (Smardon, 2016). The country has a long history of visual impact assessment guidelines, such as the Forest Service's Visual Management System (USDI Bureau of Land Management, 1980), the Federal Highway Administration's visual resource guidance document for highway projects (US DOT/FHWA, 1988), and the Foundations for Visual Project Analysis (Smardon et al., 1986). There have been significant developments in the area of visual impact assessment in Europe too, especially the United Kingdom. As per EU Directive 2011/92/EU, as amended by 2014/52/EU (European Union, 2017), potential visual impacts due to architectural characteristics of a development must be assessed during the screening process of environmental impact assessment. Visual impact assessment has been standardized in the United Kingdom through the publication of the *Guidelines for Landscape and Visual Impact Assessment* (Landscape Institute/Institute of Environmental Management & Assessment, 2002). The biggest development partner for developing countries—the World Bank—provides directive through an overarching document *Environmental and Social Framework*, in addition to its specific impact assessment guidelines for the numerous developing countries in Asia, Africa, and Latin America that receive its development funds. In this document, visual impacts are considered a form of pollution (World Bank, 2017). Finally, China, one of the world's biggest economies, also has the provision of visual impact assessment under the guidance of the *Environmental Impact Assessment of Construction Projects* (Government of the People's Republic of China, 2011).

## IMPACTS OF NATURAL LANDSCAPE LOSS ON HUMAN EMOTION AND BEHAVIOR

Over 50% of the world's human population resides in cities, and by 2050, it is estimated that 68% of people will live in urban centers (UN DESAPD, 2018). People grow up and live increasingly segregated from the natural world. Egan and Mullin (2017) highlight that “because climate change is an abstract and distant phenomenon, and individuals do not have experiences that can be attributed to it with certainty, the cognitive frameworks we use to process information have important bearing on the perceptions we form.” Technological isolation from raw nature in the digital age similarly acts as a disconnect and is “triggering emotional death,” whereby the natural world is ignored and humans no longer react to its loss (Albrecht, 2019, p. 67).

Pro‐environmental behavior comes in many forms—from what people purchase and eat to how they spend their leisure time and whom they vote for (Abrahamse, 2019). Decisions to act environmentally emerge from interactions between myriad internal and external factors, but knowledge and emotions are key. As Jane Goodall is quoted to have said: “Only if we understand can we care. Only if we care will we help. Only if we help shall all be saved” (Lindsey, 1999, p. 5). The influence of environmental knowledge and pro‐environmental emotions on environmental action is supported by empirical research (Ienna et al., 2022; Liu et al., 2020).

For people who live segregated from nature, environmental knowledge and connections to nature are mostly shaped by formal education programs, informal environmental campaigns, documentaries, and social media narratives. During these “second‐hand” experiences, people have historically only been exposed to the benefits of nature and only recently to humanity's negative impact on the environment (Jones, 2018). Even today, environmental campaigns portray both positive messages, such as the beauty of the Great Barrier Reef and tropical forests, and negative messages, such as coral bleaching and plastic pollution. First‐hand experiences of the natural world remain much less common for those people who live segregated from nature. And when these people have direct experiences with nature, they choose activities in locations that maximize positive contact with nature (e.g., camping, visiting zoos). What is so often missing in this array of environmental experiences are negative first‐hand experiences. For example, few would choose to camp within a few hundred meters of a landfill. Overtly exposing the public to environmental degradation and destruction by visually removing barriers could begin to address this experience gap, increase environmental knowledge, trigger pro‐environmental emotions, and help recalibrate people's cognitive understanding of what biodiversity loss means in reality, which all together are likely to help motivate positive environmental actions.

Several lines of research converge on the possibility that visual loss of landscapes might be an effective means of eliciting pro‐environmental cognitive and emotional understanding, even in populations who have little contact with nature. First, humans have an innate connection to the living world, referred to as biophilia (Kellert & Wilson, 1993; Wilson, 1986). People prefer natural landscapes to built landscapes, and natural landscapes have greater psychological restorative effects than built ones (Kang & Kim, 2019; Maller et al., 2006; Shanahan et al., 2016). These effects include short‐term recovery from stress or mental fatigue, faster physical recovery from illness, and long‐term overall improvement in people's health and well‐being (Velarde et al., 2007). Mental and social well‐being are now recognized dimensions of human health by the European Landscape Convention Council, and exposure to natural landscapes is recommended in healthcare provisions (Maller et al., 2006).

It follows that the physical destruction of landscapes can cause psychological distress. Loss of a landscape can elicit ecological grief associated with losing the species, the ecosystems, and the ways of life attached to that place (Albrecht et al., 2007). Grief associated with physical or ecological loss is conceptually distinguished from ecoanxiety, which emphasizes worry about anticipated future loss, and solastalgia or the loss of a cherished place (Comtesse et al., 2021). Although psychological distress can be detrimental to mental health, it can also be a normal and reasonable response to environmental loss, and quantifying its prevalence can provide an impetus for juridical change (Cunsolo & Ellis, 2018).

Psychological distress is most often experienced by those at the forefront of loss, scientists and conservation advocates who witness the disappearance directly, and people who live and work in agriculture and forestry or have strong cultural attachments to it (Comtesse et al., 2021; Cunsolo & Ellis, 2018). This vulnerability supports the suggestion that direct exposure to ecological loss is a powerful psychological trigger. Technological advances are providing unprecedented access to visual information of environmental degradation in real time, which is increasingly being used by environmental organizations to garner support. Alongside communicating this “second‐hand” imagery to the broader public, overtly exposing environmental degradation and destruction by removing visual barriers to environmental destruction would allow people to see humanity's impacts firsthand. Merely seeing environmental destruction is likely to have a strong psychological impact.

Overtly displaying environmental degradation and destruction might also help form a concrete representation of what biodiversity loss means. Indeed, although biodiversity can easily be associated with imagining animals going about their natural lives in intact natural environments, creating a concept of biodiversity loss as an absence of diversity is more challenging. Allowing people to observe the contrast between intact and destroyed or degraded environments if they are no longer hidden might help them concretize what loss means. Overtly exposing the public to environmental destruction and degradation might also help elicit environmental action in response to these human impacts on the nonhuman world.

## BENEFITS OF THE CURRENT SYSTEM

Although we stress the problems associated with concealing environmental degradation, there may also be some benefits. Planted tree screens along major roads and property boundaries that provide a visual barrier may become the only form of native vegetation remaining in the landscape. In agricultural landscapes, this may provide connectivity between larger patches (Magioli et al., 2016). We consider these benefits minor compared with those that are likely to arise when the general public can see the impacts humanity is having and then can no longer accept them.

## POLICY RECOMMENDATIONS

Adopting behaviors that avoid environmental destruction and selecting political leaders who are environmentally engaged are central to addressing the current biodiversity extinction crisis. Concealing environmental destruction limits society's comprehension of the scale of degradation, so we recommend that the concealment of environmental degradation should cease. Although some environmental screens may provide important refuges and habitat connectivity benefits, society deserves to see the full scale of the environmental devastation that is occurring around them. Living in veiled ignorance has facilitated the biodiversity crisis (Pimm & Raven, 2000 ), and Earth faces death by a thousand cuts (lingchi [凌遲]) (Shen, 2006).

Despite the development of so many guidelines for visual impact assessment that seek to safeguard landscape value and visual amenity, there remain challenges in ensuring that visual impact assessments are adequately assessed and appropriate mitigation measures are adopted. This is because visual impact assessment is largely a qualitative and subjective assessment process (del Carmen Torres‐Sibille et al., 2009). It cannot quantify impacts; rather, potential impacts are assessed and expressed in qualitative terms, such as high, moderate, and minor impact. This qualitative nature can create confusion among the decision‐makers and the community and provides room for manipulation. Without stringent guidelines, assessment has a high chance of being biased by the authors based on their age, education, and interests (Beer et al., 2023). We argue that visual barriers to hide the ugliness of development provide a false sense of security in the community in relation to environmental protection.

The application of integrated greening of gray infrastructure illustrates how multifunctional structures can provide biodiversity benefits and illustrates the risk of misuse and greenwashing (Firth et al., 2020). Unless society can directly see the environmental impacts occurring around them, they are unlikely to care or act. As heritage assessments can photograph and record historically important items, conservationists should be showing the public what the landscape originally looked like. For example, pictures and maps of areas prior to major anthropogenic change should be presented at rest stops along highways, viewing areas of mines, headlands overlooking offshore windfarms, and forestry zones inter alia.

Our work points to several policy and future research needs. First, how can the impact of public images of damaged landscapes be maximized? Afterall, concealing the scale of environmental degradation from society has not worked to date. Second, are there other artistic and creative media that can communicate environmental destruction in meaningful ways? Third, how can increasingly displaying environmental destruction avoid habituation? We hope our essay will encourage policy makers to consider halting the concealment of environmental destruction from the public and researchers to tackle these important questions.

## Supporting information



Supporting Information
